# Investigating the role connective tissue fibroblasts play in the altered muscle anatomy associated with the limb abnormality, Radial Dysplasia

**DOI:** 10.1111/joa.14040

**Published:** 2024-04-16

**Authors:** George R. F. Murphy, Eleanor Feneck, James Paget, Branavan Sivakumar, Gill Smith, Malcolm P. O. Logan

**Affiliations:** ^1^ Randall Centre of Cell and Molecular Biophysics King's College London London UK; ^2^ Plastic and Reconstructive Surgery Department Great Ormond Street Hospital for Children London UK; ^3^ Targeted Therapy Team, Chester Beatty Laboratories Institute of Cancer Research London UK

**Keywords:** connective tissue fibroblasts, extracellular matrix, limb defects, muscle bundle, radial dysplasia

## Abstract

Radial dysplasia (RD) is a congenital upper limb birth defect that presents with changes to the upper limb anatomy, including a shortened or absent radius, bowed ulna, thumb malformations, a radially deviated hand and a range of muscle and tendon malformations, including absent or abnormally shaped muscle bundles. Current treatments to address wrist instability caused by a shortened or absent radius frequently require an initial soft tissue distraction intervention followed by a wrist stabilisation procedure. Following these surgical interventions, however, recurrence of the wrist deviation remains a common, long‐term problem following treatment. The impact of the abnormal soft connective tissue (muscle and tendon) anatomy on the clinical presentation of RD and the complications following surgery are not understood. To address this, we have examined the muscle, fascia and the fascial irregular connective tissue (ICT) fibroblasts found within soft connective tissues, from RD patients. We show that ICT fibroblasts isolated from RD patients are functionally abnormal when compared to the same cells isolated from control patients and secrete a relatively disordered extracellular matrix (ECM). Furthermore, we show that ICT fibroblast dysfunction is a unifying feature found in RD patients, even when the RD clinical presentation is caused by distinct genetic syndromes.

## Graphical abstract

Type III collagen is abundant in the endo and perimysium of control patient muscle (A and E) but is downregulated in RD muscle (I and M).

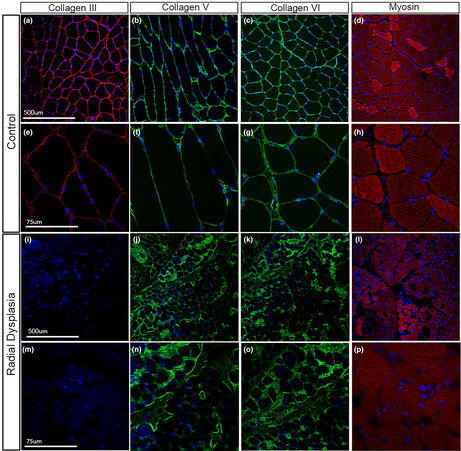

## 1 INTRODUCTION

Radial dysplasia (RD) is a disfiguring and disabling congenital upper limb anomaly with a frequency of 1 in 6000–8000 live births, with perinatal mortality often resulting prior to birth (Ekblom et al., 2010; Giele et al., 2001; Koskimies et al., 2011). The severity of deformity can vary considerably but upper limb defects often include a shortened or absent radius, bowed ulna, radial deviation of the hand, thumb malformations, and can occur unilaterally or bilaterally. There are also a range of muscle and tendon defects, including absent or abnormally shaped muscle bundles. Currently, there is no definitive cure and the optimum treatment strategy is actively debated, involving both surgical and non‐surgical methods depending on clinical presentation (Murphy et al., 2017). Severe hypoplasia or the more commonly seen total absence of the radius result in an unstable and deviated wrist joint, which leads to significant functional impairment and cosmetic concern. The aim of surgery is to create stable realignment of the carpus and hand on the distal ulna, improve function of the limb as a whole and maximise growth potential. This is achieved through an initial soft tissue distraction procedure followed by surgery to create a stable wrist construct with minimal pressure on the growing ulna physis. Soft tissue distraction aims to minimise the need to further shorten an already hypoplastic limb, reduces pressure on the growing ulna, optimises chances of preserving ‘wrist’ (ulna‐carpal) mobility and preventing recurrent deformity. Despite these efforts recurrence of radial deviation and poor forearm growth is common among patients that undergo surgery (Murphy et al., 2017). To improve treatment strategies and reduce recurrence rates a firmer understanding of the underlying cellular and molecular mechanisms that contribute to the development of the soft tissue phenotype in RD is needed.

The clinical presentation of RD is associated with several, distinct genetic syndromes. Holt‐Oram syndrome (HOS) is characterised by deformities in the upper limb, including RD combined with heart defects. HOS is associated with autosomal dominant mutation in *TBX5* (Guo et al., 2016; Li et al., 1997; Mori & Bruneau, 2004; Sun et al., 2004). Other syndromes that include RD as part of their characteristic presentation include VACTERL (vertebral, anal, cardiac, tracheo‐oesophageal, renal and limb defects) X and H, which are associated with the genes *ZIC3*/*FANCB* and *PTEN*, respectively (Chen et al., 2016), thrombocytopenia‐absent radius syndrome (TAR), associated with compound mutations in *RBM8A* and a regulatory single nucleotide polymorphism (SNP) (Albers et al., 2012), FANCONI anaemia, associated with multiple mutations and Townes‐Brocks, associated with mutations in *SALL1* (Kohlhase, 2000). Rarer syndromes that can present with RD include Baller‐Gerold, Rothmund‐Thompson, Duane‐radial ray/Okihiro and Lacrimo‐auriculo‐dento‐digital (LADD) that are associated with mutations in the genes *RECQL4*, *SALL4*, *FGFR2*, *FGFR3* and *FGF10* (Kohlhase et al., 2003; Liu, 2010; Rohmann et al., 2006; Van Maldergem et al., 2006). Some of these syndromes include mutations specifically in a fibroblast growth factor (FGF) (FGF10) and FGF receptors (Elmakky et al., 2015). Environmental insults can also cause RD, with some cases linked to maternal exposure of teratogenic drugs, including valproate, phenobarbital, aminopterin and thalidomide (Oshima et al., 2006; Ylagan & Budorick, 1994). How the pathways from all these causative genetic and environmental factors converge to produce the same diagnostic limb phenotypes to be classed as RD, remains unclear. Therefore, targeting the primary cellular mechanisms that contribute to RD aetiology and disease progression is required.

During embryogenesis, irregular connective tissue (ICT) fibroblasts, sometimes referred to as muscle connective tissue (MCT), orchestrate the process that organises muscle progenitors into discrete muscle bundles (Hasson et al., 2010; Kardon et al., 2003; Mathew et al., 2011) and this may be mediated by the extracellular matrix (ECM) they secrete (Besse et al., 2020). Conditional knockout of *Tbx5* in mouse ICT fibroblast cells disrupts muscle bundle formation and alters the overall size and shape of individual muscle bundles, sometimes leading to their absence (Besse et al., 2020). These studies demonstrate functional ICT fibroblasts are necessary to communicate to the muscle cells to organise their arrangement into muscle bundles.

Understanding the role of human ICT fibroblasts in RD remains unexplored and revealing any disruption to this cell population could contribute to understanding RD aetiology and developing future treatments. To address this, we have examined the muscle, fascia and the fascial irregular connective tissue (ICT) fibroblasts found within soft connective tissues from RD patients. This study investigates intrinsic changes to limb connective tissue fibroblasts in RD patients as a potential cause of the disease phenotype and contribution to post‐distraction recurrence. By establishing primary cell lines from RD and control patients and analysing their properties, we show that ICT fibroblast dysfunction is a unifying feature found in RD patients, even when the RD clinical presentation is caused by distinct genetic syndromes.

## 2 RESULTS

### 2.1 Establishment of human ICT fibroblast cell lines from radial dysplasia (RD) and control patients

**FIGURE 1:**
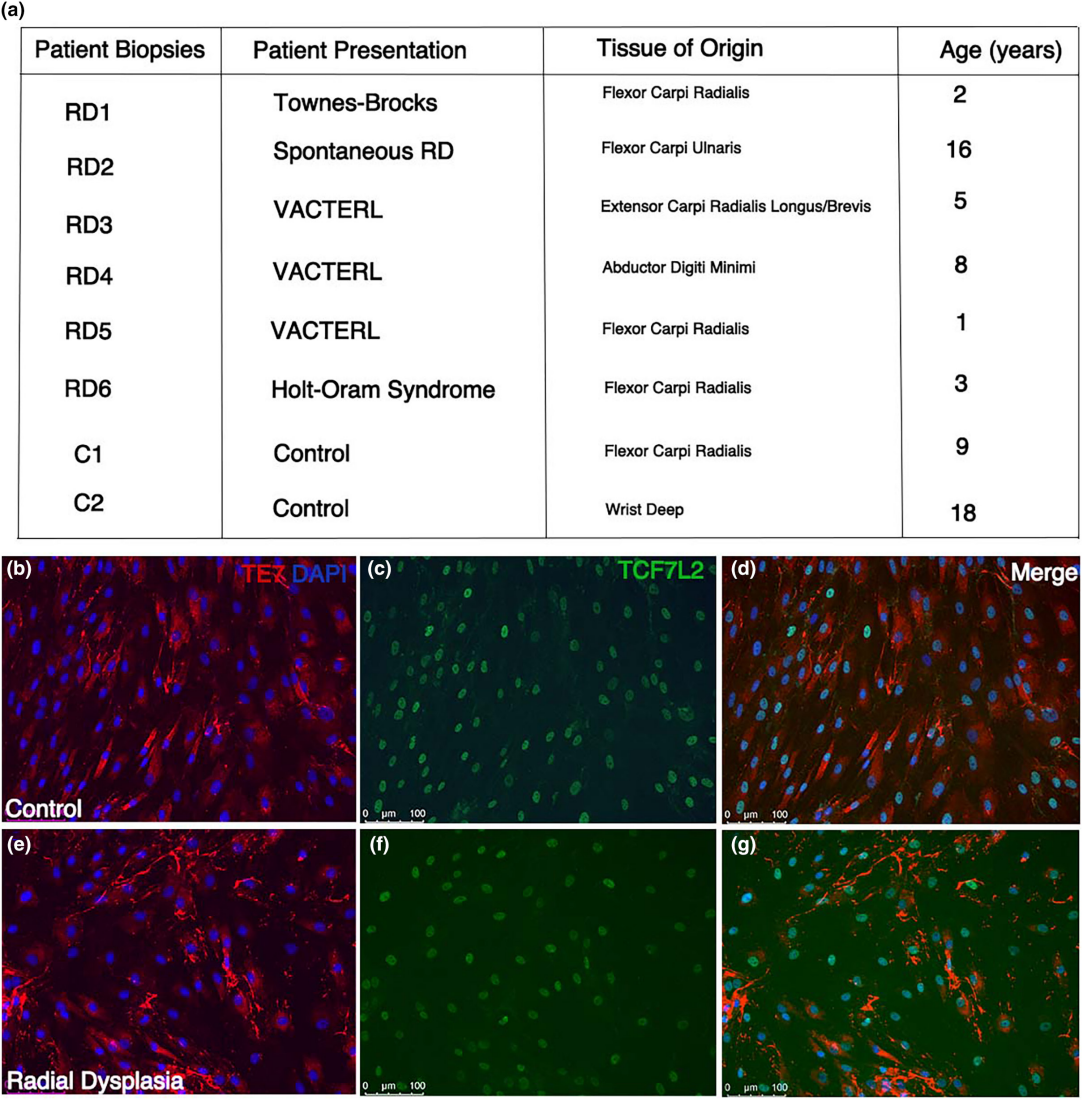
Connective tissue fibroblast characterisation. Patient biopsy samples are listed alongside the patient presentation and the tissue of origin that the biopsies are obtained (a). Control (b–d) and radial dysplasia (e–g) patient connective tissue cells are characterised using targeted fibroblast (TE7) and muscle connective tissue (TCF7‐like 2) antibody staining. Most of the cultured cells from the patient biopsies highly express TE7 and TCF7‐like 2, suggesting the population of cells are connective tissue fibroblasts (b–g).

To explore the potential abnormalities in the cellular and molecular properties of ICT fibroblast cells in RD patients, we obtained tissue biopsies from control and RD patients undergoing surgery at the wrist and hand. Biopsies were mostly obtained from the radial side and contained fascia and muscle, usually from the flexor carpi radialis muscle (Figure 1a). A total of 18 control and RD patients were recruited during the study, and we were able to derive stable primary cell lines from six of the RD patient tissue biopsies. Some of the initial samples were not processed to generate cell lines and other patient samples had different conditions that did not include RD. If tissue biopsies were sufficiently large, they were divided, and one portion was dissociated to obtain isolated cells while the remaining portion was retained for histological analysis. Biopsies were obtained from a spectrum of RD patients with a range of different genetic diagnoses, including Holt‐Oram syndrome, VACTERL and Townes‐Brocks (Figure 1a).

The patient tissue biopsies could have also contained other cells found around muscle fascia including muscle cells, tendon and adipose cells. We predicted, however, that following expansion of the cell lines, the post‐mitotic muscle cells would fail to expand and would be lost, and the fibroblasts would rapidly become the predominant cell type. To confirm the identity of the cells obtained in the primary cell lines, human fibroblast and connective tissue cell markers were employed to confirm that the cultures contain ICT fibroblasts (Figure 1b–g). We found cultures were predominately a fibroblast dense population at passage number 2 after tissue dissociation. The TE7 antigen is a well‐established marker for mesoderm‐derived human connective tissue fibroblasts in tissue sections and in cell culture (Haynes et al., 1984). TE7 is detected in the cytoplasm of the dissociated cells taken from the RD and control patient biopsies (Figure 1b,e). In addition, TCF7‐L2 (previously known as TCF4) is a nuclear marker of human connective tissue cells and is routinely used as a marker of the muscle connective tissue fibroblast population (Kardon et al., 2003). We detect uniform expression of TCF7‐L2 in cell cultures (Figure 1c,f), suggesting that our cultures represent a highly enriched population of irregular connective tissue/muscle connective tissue fibroblasts.

**FIGURE 2:**
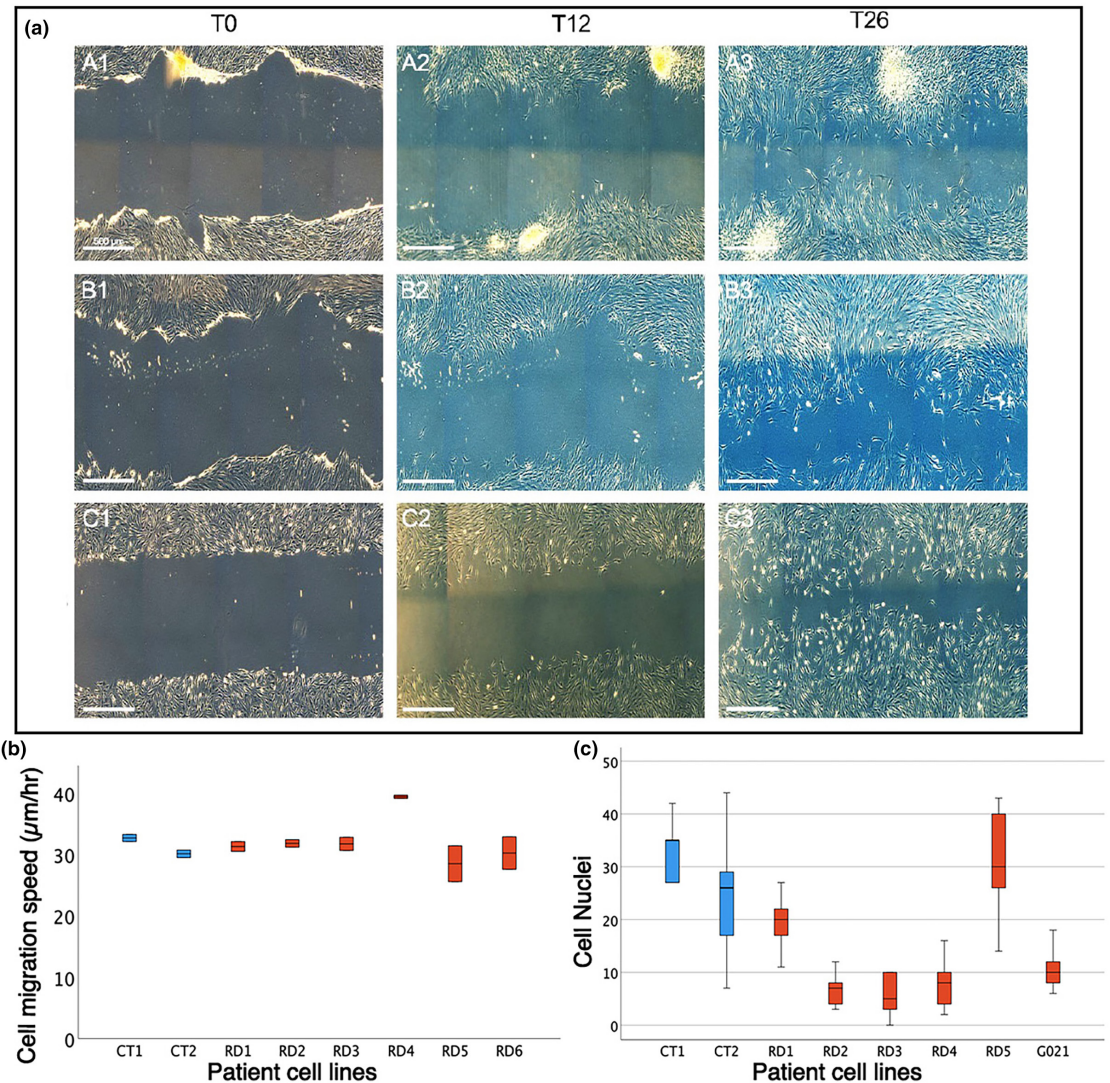
Scratch cell migration and cell adhesion assays. Cell migratory speeds were calculated over a 26‐h period from scratch formation, measured at 5 points in two wells per cell line. (a1–a3) images show control cell migration (a). RD1 (c1–c3) and RD6 (b1–b3) fibroblast cell migration (a). Boxplot of mean cell migration speed (b). Boxplots of cell counts by cell line for cell adhesion assay of control (c‐shown in blue) and radial dysplasia (RD‐shown in red) cell lines (c).

Fibroblast cells in culture have a characteristic branched, stellate or elongated morphology, readily adhere to and migrate on tissue culture substrates and proliferate freely until they reach confluence and exhibit contact inhibition (Abercrombie, 1978). To compare these features between control and RD ICT fibroblasts, we carried out two assays to quantify these core properties of fibroblasts. In adhesion assays (see Materials and Methods), all RD and control cell lines demonstrated an equivalent ability to adhere to the tissue culture dish, with no significant difference in substrate adherence properties among the cells analysed using a one‐way ANOVA and a post hoc Tukey test (Figure 2c). To determine any cell migration differences between control and RD cell lines a migration assay was conducted, and speed of cell movement calculated by measuring the distance between the scratch edges at five fixed points over three different time points within 26 h (Figure 2a,b See Materials and Methods). In the migration assays, we observed that cells from each individual line migrate at a similar speed. RD4 cells migrated slightly faster than the other cell lines, but this was not statistically significant (*p* = 0.13) when a one‐way ANOVA and a post hoc Tukey test was performed. Overall, all cell lines demonstrated equivalent abilities of substrate adherence and migration. We also did not detect any difference in rates of cell proliferation in any of our cultures, but this was not quantified.

### 2.2 RD ICT fibroblasts secrete a relatively disorganised extracellular matrix

**FIGURE 3:**
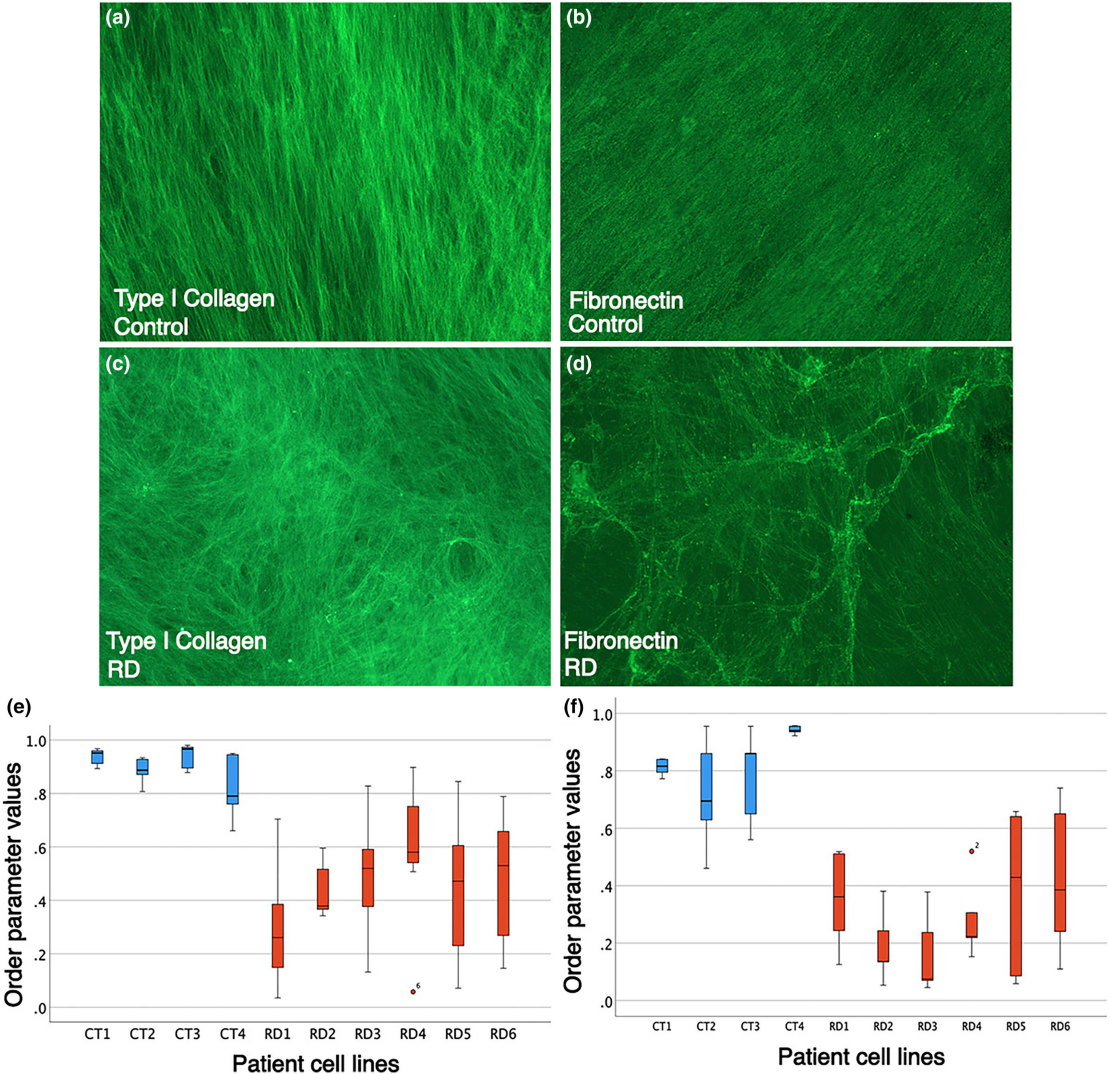
Radial dysplasia connective tissue cell‐derived extracellular matrix is significantly disorganised. Connective tissue cell‐derived extracellular matrix synthesised from control (a, b) and radial dysplasia (RD) (c, d) patient cells are stained with type I collagen and fibronectin. Box and whisker plot of order parameter values demonstrating RD cell line derived matrix (*n* = 6 different RD patient‐derived cell lines) is significantly more disorganised compared with the control cell line derived matrix (*n* = 4 different control cell lines) when stained for type I collagen (e) and fibronectin (f) fibres. Control, blue. RD, red.

The extracellular matrix (ECM) of muscle tissue, which is secreted primarily by the ICT fibroblasts, has important roles in muscle development, homeostasis and regeneration (Kjaer, 2004; Purslow, 2020). To test whether there are any differences in the ECM synthesised by control and RD ICT fibroblasts, we generated cell‐derived matrices (CDMs) to qualitatively assess the matrix they secrete. Type I collagen and fibronectin are the two primary constituents of the ECM. We therefore analysed these matrix components to assess the organisation of the CDMs. Type I collagen secreted by the control ICT fibroblasts has a regular ordered arrangement of fibres (Figure 3a). In contrast, the type I collagen staining pattern in matrix secreted by RD ICT fibroblasts is more disorganised (Figure 3c). Similarly, fibronectin has a regular ordered alignment of fibres in control ICT fibroblast CDM compared with a more disorganised array in RD samples (Figure 3b,d). In addition, the staining pattern in RD CDM appears more punctate, with areas of increased fibronectin density compared to the control CDM, where the fibronectin fibres appear more linear and uniform (Figure 3b,d). No apparent changes in fibronectin and type I collagen expression levels were detected among the control and RD patient cell lines analysed (Figure 3a–d).

To quantify the differences of the type I collagen and fibronectin fibres organisation, we employed a method where images are analysed for fibril alignment using a MATLAB script (Cetera et al., 2014). The script works by dividing each image into a grid, calculating the mean vector of any linear structures within each grid square and then summing the relative alignment of vectors to a single number (order parameter) per image, between 0 (random alignment) and 1 (perfect alignment). Calculated order parameters confirm that the control patient CDM is significantly more organised compared with the less ordered RD cell‐derived matrix when pairwise comparisons were conducted with a post hoc Tukey test (Figure 3e,f). Type I collagen (*F* = 9.918, *p* < 0.001) and fibronectin (*F* = 39.759, *p* < 0.001) are significantly more disorganised in the RD ICT secreted CDM compared with the control‐derived CDM. These results demonstrate that the RD ICT fibroblasts lack the ability to produce a normal extracellular matrix with a structured lattice organisation. This is consistent with the clinically observed loss of organisation in the soft tissues in many patients with RD (Buck‐Gramcko, 1985; Murphy et al., 2017).

### 2.3 Muscle cells are less organised when grown on radial dysplasia (RD) extracellular matrix

**FIGURE 4:**
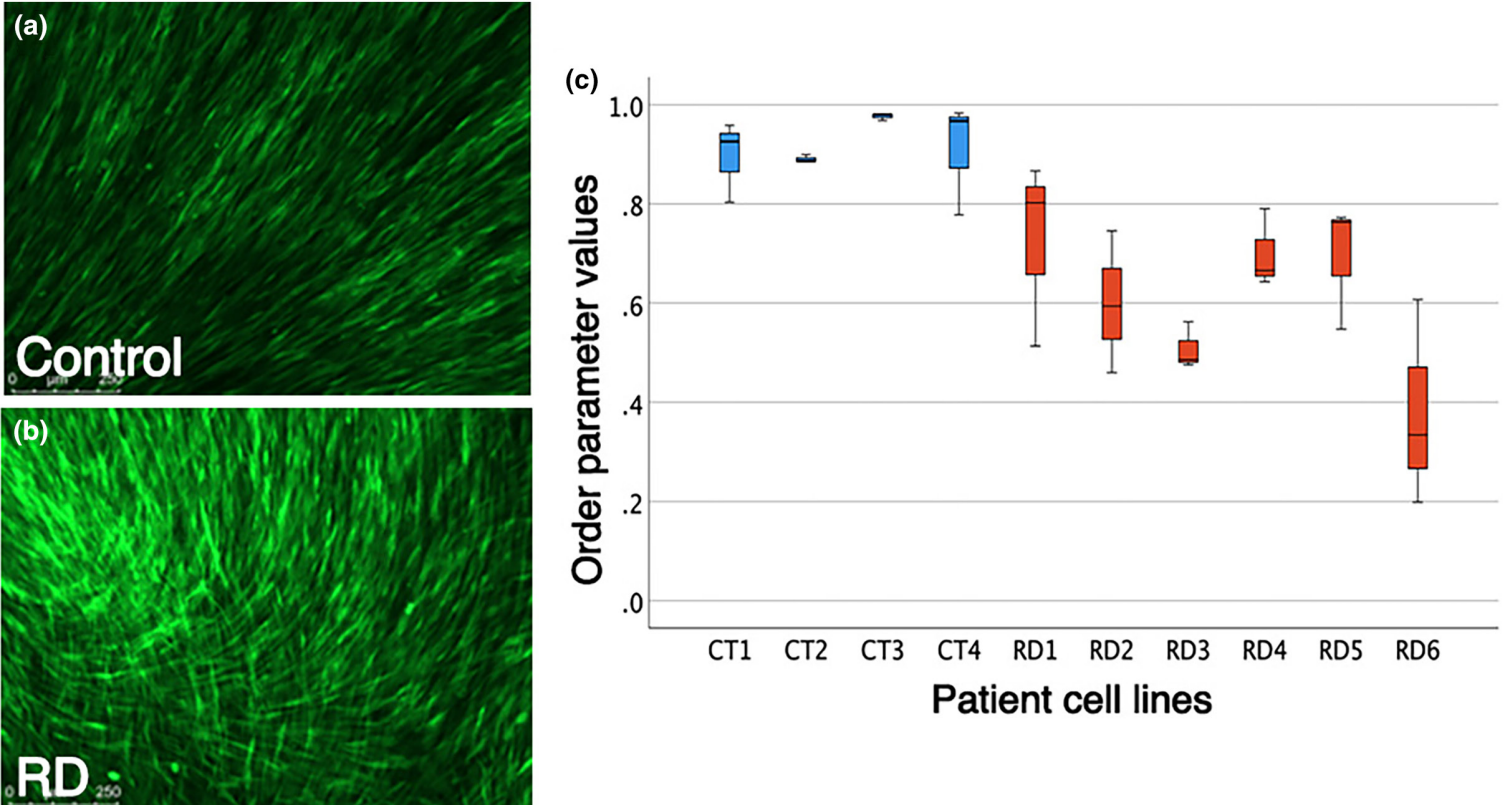
Extracellular matrix secreted by connective tissue fibroblasts can influence the alignment of muscle cells. Representative examples of the results of plating muscle cells (HMC25 line labelled with GFP) on connective tissue fibroblast‐derived matrix produced by control (a) or radial dysplasia (RD) (b) patient cell lines. A box and whisker plot of order parameter values demonstrating muscle cells are significantly less aligned when seeded on RD cell line (*n* = 6 different patient cell lines) derived matrix compared with when grown on control patient cell line (*n* = 4 different cell lines) derived matrix (c). Control, blue. RD, red.

CDMs can also be used to functionally test the behaviour of cells (Fitzpatrick & McDevitt, 2015). We wanted to examine whether control muscle cells are impacted by the two different types of matrices. We produced CDMs from control and RD ICT fibroblasts and then plated a human muscle cell line (HMC25) (GFP‐labelled using a lentivirus) onto the CDMs from each cell line to understand how the change in the extracellular matrix organisation affects the organisation of muscle cells. The lentiviral GFP labelling enabled muscle cells to be identified during live imaging, before fixation. The muscle cells plated onto control CDM are ordered and align with adjacent cells (Figure 4a). In contrast, muscle cells plated on the RD CDM are less organised compared with the control assays with muscle cells lying perpendicular to adjacent muscle cells and appearing to form a swirl pattern compared with the more linear arrangement of muscle cells seeded onto the control ICT CDM (Figure 4b). The alignment of the muscle cells was quantified following the same method used to measure CDM organisation (Figure 3d,e) (Cetera et al., 2014). The calculated alignments demonstrate the muscle cells plated onto the RD CDMs are significantly more disorganised and misaligned compared with the same muscle cells plated onto control CDMs (Figure 4c). Together, these data show that muscle cells are unable to organise as they would normally, when plated onto the more disorganised substrate of CDM produced by RD patient‐derived ICT fibroblasts.

### 2.4 Composition of the extracellular matrix

**FIGURE 5:**
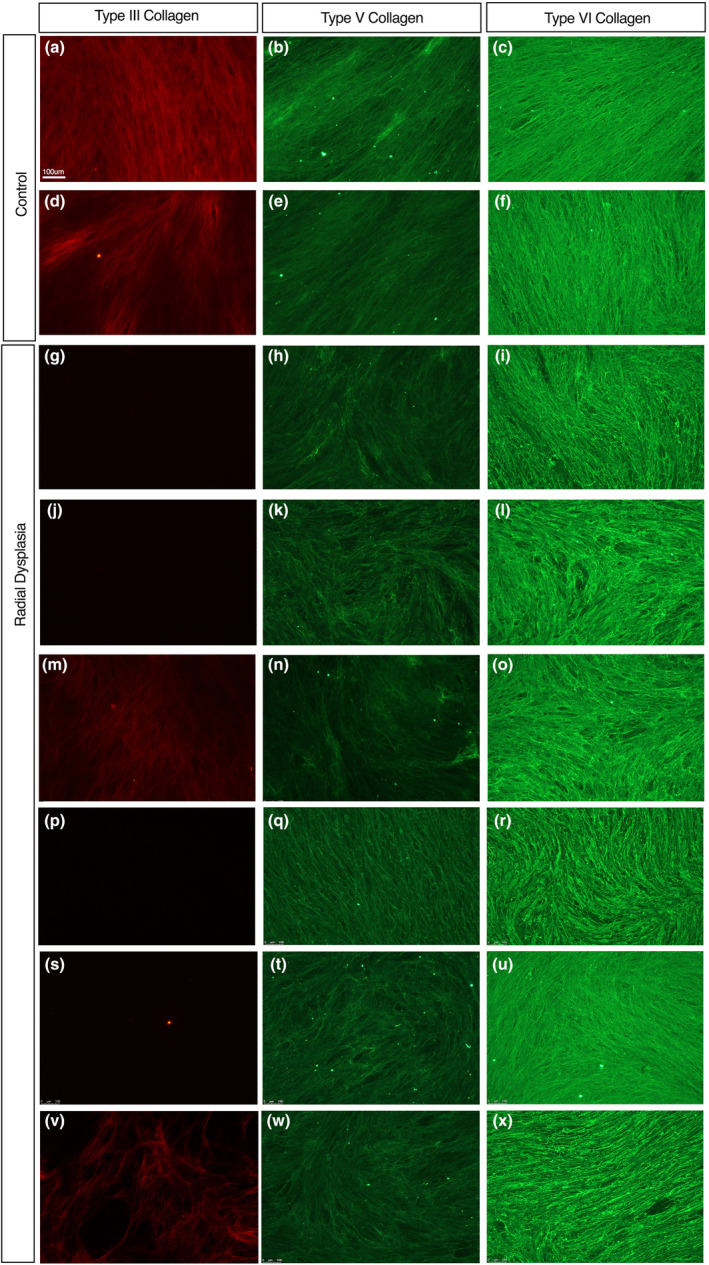
Type III, V and VI collagen expression in connective tissue fibroblast‐derived extracellular matrix. Patient cell‐derived extracellular matrix from control patients (a–f) positively stained with type III (a, d), V (b, e) and VI (c, f) collagen in all cell‐derived matrices analysed. Radial dysplasia cell‐derived matrices (g–x) stained positively for type V (h, k, n, q, t and w) and VI collagen (l, o, r, u and x). Type III collagen was significantly downregulated or absent radial dysplasia cell‐derived matrix (g, j, m, p, s and v) when compared to the control type III collagen‐stained matrices (a, d).

The CDM assays demonstrate there are organisational and functional differences in RD extracellular matrix. To determine whether there are any qualitative differences in the matrix secreted by RD ICT fibroblasts, we analysed the composition of the extracellular matrix. We did not detect any quantitative changes in the two most common extracellular matrix components, type I collagen and fibronectin, secreted by the ICT fibroblasts (Figure 3). We therefore looked at other extracellular matrix components known to be expressed in muscle ICT (McKee et al., 2019). Type III collagen was markedly downregulated, with no or little type III collagen detectable in the RD‐conditioned extracellular matrix samples (Figure 5g,j,m). There was also a more subtle yet reproducible reduction in type V collagen in the RD CDM samples; however, we did not detect differences in staining intensity of type VI collagen in any of the RD samples analysed (Figure 5). In summary, the data show that RD ICT fibroblasts are producing normal amounts of many of the normal matrix constituents but that there is a reduction in expression of some, specific ECM components.

**FIGURE 6:**
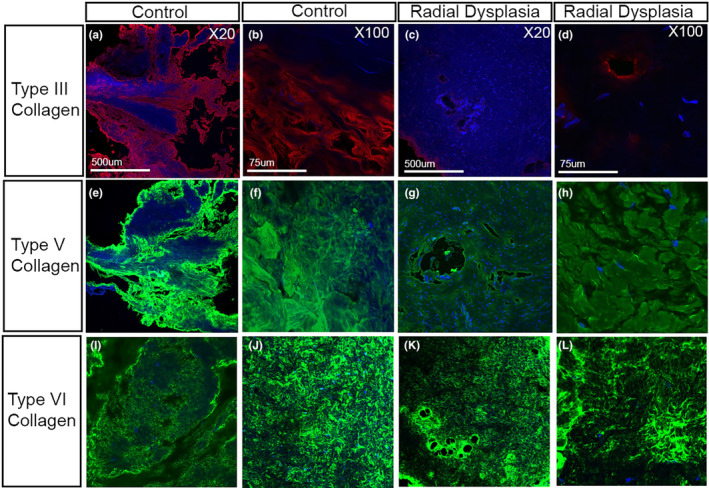
Type III, V and VI collagen expression in patient fascial biopsies. Fascial biopsies taken from control and radial dysplasia (RD) patients were analysed for type III (a–c), V (e–g) and VI (i–k) collagen. Type III collagen was downregulated in the immunofluorescent stained RD samples compared to controls. Type V collagen showed a slight downregulation of staining within the RD connective tissue biopsies compared to controls (e–g) with type VI collagen (i–l) showing a similar staining in the control and RD biopsies processed.

**FIGURE 7:**
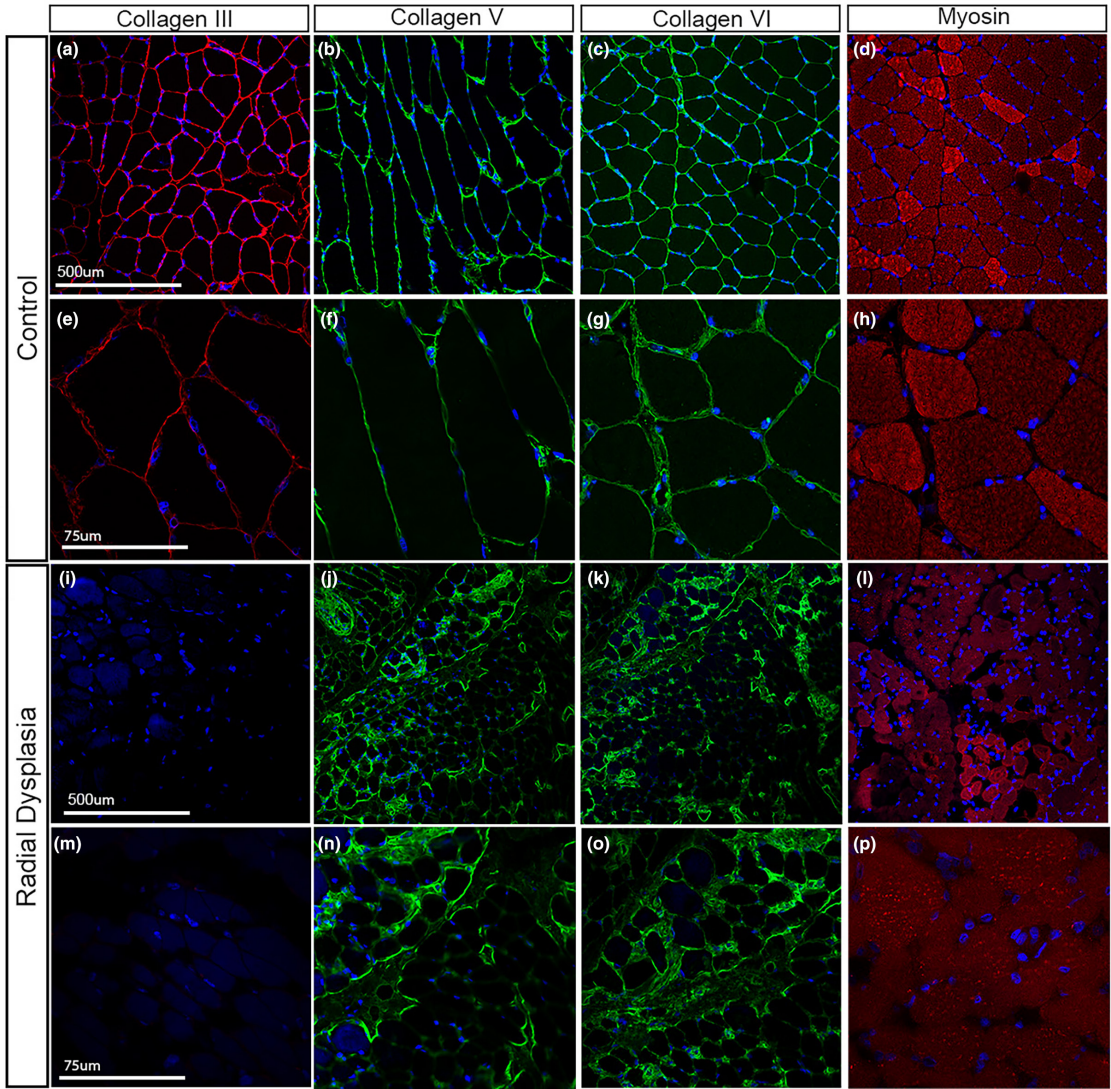
Type III, V and VI collagen expression in patient muscle biopsies. Control (a–h) and RD (i–) muscle biopsies analysed with collagen III, V, VI and myosin at X20 (a–d, i–l) and X40 (e–h, m–p) magnifications. Type III collagen is abundant in the perimysium of control patient (a,e) but is downregulated in the RD muscle sections (i, m). Type V and VI collagen is abundant within the perimysium of all muscle samples (b–c, f–g, j–k, n–o). Myosin (d, h, l, p) positivity (red) merged with DAPI (blue) shows the muscle structure of the samples (d, h, l, p).

To confirm that this reduction in type III collagen expression in the cell culture environment was replicated in vivo, immunofluorescence staining was carried out on muscle and fascial tissue patient biopsy samples (Figures 6 and 7). Type III collagen was reduced in the RD fascial samples compared with the control samples (Figure 6a–d). There was also a noticeable reduction in type V collagen staining (Figure 6e–h). In contrast, there was no clear change in type VI collagen staining (Figure 6i–l). An RD muscle biopsy sample revealed a similar staining pattern (Figure 7). Type III collagen was downregulated within the RD muscle biopsy compared with the control (Figure 7a,e,i,m), while there was little or no apparent change in the type V and type VI collagen distribution (Figure 7b,c,f,g,j,k,n,o). Specifically, type III collagen was found to be reduced within the perimysium layers, that surround the muscle fascicles, in the RD muscle biopsies sample (Figure 7i,m).

## 3 DISCUSSION

Our results show that the extracellular matrix (ECM) secreted by ICT fibroblasts from RD patients is disorganised compared with matrix secreted by the equivalent cells isolated from control patients. We further show that this relative lack of matrix organisation has a functional impact on the ability of ICT fibroblast cells to support alignment of muscle cells on a matrix. Our data also demonstrate that muscle cells plated onto RD matrix are disorganised in response to the matrix substrate, and that the muscle cells were unable to correct or realign the matrix to produce a more ordered alignment of fibres in culture. These results are consistent with a model in which an inherent defect in the ICT fibroblasts of RD patients is directly responsible for the abnormalities in limb soft tissues (muscle and tendons) characteristic of this limb defect. These soft tissue defects can include but are not limited to, absence of individual dorso‐radial muscles, tendons and connective tissues, as well as a loss in normal muscle tissue architecture and muscle bundle anatomy such that a single smaller dorso‐radial muscle mass exists, frequently with similar tendon insertions, but where the individual muscles are conjoined and indistinguishable as discrete entities. This model is also consistent with our observations in a mouse phenocopy of HOS, where we have shown that conditional deletion of *Tbx5* in ICT fibroblasts, but not muscle or tendon cells, can cause abnormal muscle and tendon formation similar to that seen in HOS (Besse et al., 2020; Hasson et al., 2010). The data we report here, using cells isolated from a cohort of RD patients with a range of different genetic syndromes, demonstrate that defects in the matrix secreted by ICT fibroblasts can also explain the aetiology of the limb soft tissue abnormalities of other syndromes such as TAR, VACTERL and Townes‐Brocks, which can all present with clinical features of RD. This suggests that a defect in the ICT fibroblasts and the matrix they secrete is a unifying feature of the different genetic syndromes that present with RD soft tissue abnormalities. We provide the first study that demonstrates a defect to the connective tissue fibroblasts and the matrix they secrete. A previous publication has proposed that soft tissue defects are not a driver of the radial dysplasia phenotype and have not observed any change to the histology of radial dysplasia muscle (Mittal et al., 2022). This study did not assess the connective tissue fibroblasts in muscle.

The ECM is a dynamic network of various macromolecules that is continuously remodelled in response to surrounding stimuli. The extracellular matrix provides cells with structural support and can influence environmental signals through modulation of signalling cascades and growth factors to maintain tissue homeostasis. A loss of ECM organisation or constituent matrix proteins underlies many diseases. In mature tissues, any impairment to the remodelling of the extracellular matrix can lead to fibrosis or scarring, osteoarthritis and cancer progression (Bonnans et al., 2014; Iredale et al., 2013; Radisky et al., 2002). In many of these pathologies, specific matrix proteins have been highlighted to be up‐ or downregulated that has a resultant knock‐on effect on downstream proteins and signalling pathways that contribute to the overall disease aetiology. In several of these cases, the principal matrix component has been targeted for treatment strategies. For example, natalizumab is an effective therapy in multiple sclerosis and Crohn's disease that targets cell adhesion‐promoting integrin subunits to prevent leucocyte migration through the matrix substrate (Kümpfel et al., 2002).

We show that type III collagen is downregulated or absent in the RD cell‐derived matrices. Furthermore, we show type III collagen is markedly downregulated in the perimysial component of the myofascial layers of RD muscle biopsies. The perimysium surrounds the muscle fascicle and enables the transmission of contractile movement laterally. This myofascial layer is abundant in type I collagen, with smaller quantities of type III, V and VI collagen (Purslow, 2020). Type III collagen is a fibril‐forming collagen abundantly expressed in tissues, including skeletal muscle, blood vessels and cornea that require structural support to withstand the exerted forces they experience. Type III collagen has been assigned a range of functions within the extracellular matrix to support surrounding cells and matrix components for the development, maintenance and wound healing of tissues (Gay et al., 1978). The presence of type III collagen has been shown to be necessary for type I collagen fibrillogenesis, specifically for controlling the diameter of type I collagen fibres (Liu et al., 1997). In mice deficient of type III collagen, many tissues including the cardiovascular and skin elements fail to develop and function correctly due to their diminished extracellular matrix architecture (Liu et al., 1997). Although the perimysial enrichment of type III collagen is striking, the exact function of this collagen isoform in this tissue location is unknown and therefore the functional significance of its absence/reduction in RD patient muscle remains unclear. Further studies are needed to understand the effect of a reduction of type III collagen in muscle and how this may contribute to RD aetiology, muscle function in RD patients and their response to treatment.

Mutations in the *COL3A1* gene, which encodes the pro‐alpha1 chain of type III collagen, are associated with the connective tissue disorder vascular Ehlers–Danlos syndrome (EDSVASC, EDS IV, OMIM130050). Symptoms of EDSVASC mainly affect the type III collagen‐rich blood vessels, with patients suffering bruising and visible vasculature, while in more severe cases there can be arterial and bowel ruptures (Cortini et al., 2016). The joint hypermobility and skin laxity characteristic of most other forms of EDS are largely limited to the digits in EDSVASC and skin laxity is usually absent. Muscle involvement is a key feature of EDS. This can include muscle hypotonia, muscle rupture, fatigue and musculoskeletal pain (Voermans et al., 2009). Despite EDS and RD both having a reduction in type III collagen, the soft connective tissue phenotypes found in RD are at an apparent opposite end of a spectrum to EDS, EDS being associated with laxity or lack of elasticity and RD associated with ‘tight’ connective tissues. A simple explanation for the differences in the clinical presentation of RD and EDS patients is that EDSVASC phenotypes arise because of sensitivity to the systemic, heterozygous loss of *Col3A1*, while in the RD patients we have examined, reduction in type III collagen is isolated to the fascia and perimysium.

The connective tissue fibroblast cell lines we have analysed were derived from a spectrum of RD patients with different syndromes caused by distinct genetic lesions, with a common phenotype of congenital upper limb defects. Despite this, we observed a remarkable degree of similarity in the results obtained from the assays we employed between the RD cell lines. We show all RD cell lines produced a similar degree of disorganised ECM and reduced type III collagen production, revealing a common molecular dysfunction in patients that present with the clinical features of RD but with different genetic syndromes that underlie this presentation. The connective tissue fibroblasts and the extracellular matrix this cell population synthesises could be targeted as a treatment strategy for RD. Current treatment strategies are limited to surgical and non‐surgical methods, which have a high rate of recurrence among patients (Murphy et al., 2017). This study helps uncover the underlying molecular aetiology that contributes to RD, this may improve the development of treatment strategies in the future.

Our approach of establishing human ICT fibroblasts cell lines from patient biopsies and analysing their properties in molecular and functional assays enabled us to identify defects in the organisation and composition of the matrix that could explain the origins of the soft tissue defect in RD. In this study, we were not able to obtain tissue biopsies to isolate fibroblast cell types from other regions of the body. At this point, we are unable to distinguish whether the abnormalities we see in RD patient ICT fibroblasts are specific to this fibroblast cell type or if other fibroblast cell types are affected in RD patients. These cell lines and the assay we have established have the potential to identify the molecular and cellular defects that underlie the soft tissue abnormalities in RD and can be used to screening for factors that can reverse these defects and thereby offer therapeutic potential to improve treatments for RD.

Our results confirm that a functional defect in the ICT fibroblast population is part of the clinical features of RD and these directly contribute to the clinical presentation of this disease and should be appreciated in treatment strategies. We propose that defects in the ICT fibroblasts create disorganised matrix which contributes to the soft tissue abnormalities seen in RD and that these defects could explain the recurrence of radial deviation in RD patients after surgical correction. Correction of this cell type‐specific defect has the potential to improve future RD treatment outcomes.

## 4 MATERIALS AND METHODS

### 4.1 Tissue collection

Tissue collection and experiments were all carried out in compliance with the principles outlined in the World Medical Association declaration of Helsinki. The project was adopted by the UK National Institute for Health Research (NIHR) to their national research portfolio, with the Clinical Research Network ID 20411. Ethical approval was granted by the London Hampstead research ethics committee (REC reference 15/LO/2085). NHS Research and Development approval was granted from the recruiting hospitals. Tissue samples were collected with informed written parental (and where age appropriate, patient) consent.

Tissue biopsies from patients undergoing elective surgery for RD or upper limb trauma were taken during surgery. Samples used for cell culture were placed in individual sterile containers, in 0.9% sodium chloride and processed immediately after transportation by courier to Guy's Campus, King's College London. Biopsies taken for tissue analysis were flash frozen in liquid nitrogen and stored at −80°C. Specific information of the patient biopsies, the tissues site of origin and patient clinical presentation is listed in Figure 1.

### 4.2 Cell dissociation and culture

Tissue specimens were placed in sterile 2 mL Eppendorf tubes with 500 μL of whole skin dissociation kit, human reagent (Miltenyi Biotec), morcellated with iris scissors and incubated with agitation for 3 h at 37°C. The dissociated cells were triturated and plated initially into 25 cm^2^ Nunclon flasks (Thermo Scientific) with 5 mL fibroblast growth media (FGM) (Dulbecco modified Eagle's medium (Gibco, Thermo Fisher), 10% fetal bovine serum (FBS) (Gibco, Thermo Fisher), 1% penicillin/streptomycin (Gibco, Thermo Fisher), 1% GlutaMAX (Gibco, Thermo Fisher) and 1% sodium pyruvate (Gibco, Thermo Fisher)), and this was considered P0. Flasks were incubated at 37°C with 5% CO_2_, with FGM changed every 2–3 days. Cells were expanded to 70%–90% confluence prior to trypsinisation and further passaged. Highly enriched fibroblast populations were seen and characterised from passage number 2 after dissociation, this was consistent from all cell lines.

### 4.3 Fibroblast migration assay

6‐well Nunclon plates were gelatin coated as previously described (Franco‐Barraza et al., 2017), seeded with 3 × 10^5^ fibroblasts (2 wells per fibroblast line), and grown to confluence. A migration assay was then performed (Liang et al., 2007). Briefly, a transverse scratch was created in each well with a p200 pipette tip, the wells were rinsed with fibroblast growth medium and photographed on an inverted phase‐contrast microscope (Leica DMi8 microscope with a motorised stage, Leica DFC7000T camera and Leica LASX imaging software). Plates were incubated then re‐photographed at 12, 24 and 36 h. For each timepoint photographed, the distance between the scratch edges was measured at five fixed points (3, 6, 9, 12 and 15 mm from the left‐hand well side). Statistical analyses were performed using SPSS 24.

### 4.4 Fibroblast adhesion assay

Cells were grown to confluence on gelatin‐coated 6‐well Nucleon plates (Franco‐Barraza et al., 2017). Fibroblast cultures were detached with 0.05% trypsin – EDTA (Gibco), neutralised with fibroblast growth medium, and pelleted by centrifugation at 1000 RPM at room temperature for 5 min. The supernatant was aspirated, and the pellets re‐suspended in fibroblast growth medium, adjusted to 3.5 × 10^5^ cells/ml and equilibrated on a roller agitator at room temperature for 20 min. 50,000 cells (150 μL) were then plated into each well, with three wells per fibroblast line, and the plates placed in a humidified incubator for 20 min at 37°C / 5% CO_2_. Plates were then tilted, aspirated off the well side, and rinsed 3× with DPBS. Adherent cells were then fixed and nuclear stained simultaneously with 4% paraformaldehyde containing Hoechst 33342 diluted 1:500, then rinsed 2× in DPBS and imaged. Cells were counted using freeware CellProfiler software (www.cellprofiler.org, Broad Institute, Harvard/MIT). Statistical analyses were performed using SPSS 24 (IBM).

### 4.5 Immunohistochemistry of extracellular matrix, cell cultures and tissue biopsies

Cell‐derived matrices (CDM) were derived following a previously described protocol (Franco‐Barraza et al., 2017). Cell cultures were fixed with 4% paraformaldehyde (PFA) in phosphate‐buffered saline (PBS) solution for 30 min at room temperature. Tissue biopsies of muscle and fascial tissue were collected from patients and snap frozen in liquid nitrogen, cryosectioned at 10 μm and collected on Superfrost plus slides. The cell culture wells or slides were washed with PBS three times over 5 min and blocked with 5% goat serum (Gibco, Thermo Fisher), 0.5% bovine serum albumin and 0.5% Triton X‐100 in PBS for 1 h at room temperature. Primary antibodies (Table 1) were applied to the wells and incubated overnight at 4°C. The wells were further washed in PBS with 0.05% Tween‐20 (Sigma) (PBT) three times over 5 min before the secondary antibody (Goat anti‐mouse AlexaFluor 567 or Goat anti‐rabbit 488) was applied and incubated for 2 h, in the dark, at room temperature. Samples were further washed three times over 5 min with PBT before DAPI (Thermo Scientific) (diluted 1:15,000 in PBS) was added for 3 min then further washed. Stained samples were imaged with the Leica DMi8 microscope with Leica DFC7000T camera and Leica LASX imaging software or a Nikon A1 inverted confocal microscope.

**TABLE 1:** Primary antibodies.

Antibody target	Antibody species and isotype	Dilution	Company	Antibody
Type I collagen	Rabbit pc IgG	1:500	Abcam	Ab138492
Type III collagen	Mouse mc IgG1	1:500	Abcam	Ab6310
Type V collagen	Rabbit pc IgG	1:200	Abcam	Ab7046
Type VI collagen	Rabbit pc IgG	1:200	Abcam	Ab182744
Fibronectin	Rabbit pc IgG	1:200	Santa Cruz	sc‐9068
TCF7 like 2 (TCF4)	Rabbit mc IgG	1:100	Cell signalling technology	C48H11
DAPI	N/A	1:15,000	Thermo Scientific	
Myosin heavy chain (MF20)	Mouse mc IgG2b, k‐LC	1:10	Developmental studies hybridoma bank	MF20
Fibroblasts (TE7)	Mouse IgG1	1:100	Sigma‐Aldrich	CBL271

### 4.6 Extracellular matrix organisation assay

Cell‐derived matrices were derived following a previously described protocol (Franco‐Barraza et al., 2017). Nunclon 48‐well plates were coated with gelatin as previously described (Franco ‐Barraza et al., 2017), washed 3× with Dulbecco's phosphate‐buffered saline (DPBS) (Gibco, Thermo Fisher) before seeding cultured fibroblasts at a density of 25,000 cells per well and 6 wells per cell line. All cell lines were grown until confluent in FGM for 5 days and then supplemented with 50 μg/mL ascorbic acid (Sigma‐Aldrich) for a further 7 days to stimulate extracellular matrix deposition. The deposited matrices were decellularised using a solution of 20 mM ammonium hydroxide in PBS with 0.5% Triton‐X100 for 5 min at room temperature and then diluted 1:1 with PBS at 4°C overnight.

The extracellular matrix deposited by the cells was labelled immunofluorescent for fibronectin and type I, III, V and VI collagen (Table 1) following the immunohistochemistry method outlined above. Four areas of the wells stained with type I collagen or fibronectin were imaged and analysed for alignment using the MATLAB script previously described (Cetera et al., 2014). The script generated an order parameter between 1 (perfect alignment) and 0 (random alignment) for each image. All statistical analysis was performed using SPSS 24. Single channel images were analysed in ImageJ using ‘Process/Batch/Measure’ command to measure the mean grey value, as a proxy for relative extracellular matrix protein expression.

### 4.7 Generation of the GFP muscle cell line

An immortalised control human myoblast cell line, derived from the semitendinosus muscle and immortalised by retroviral co‐transduction of telomerase and cyclin dependent kinase 4, was gifted by the Zammit lab at King's College London (Mamchaoui et al., 2011). The cell line expressed both myogenic and mature myotube expression markers. Cell aliquots were modified to express green fluorescent protein (GFP) using a replication‐incompetent lentivirus. The cells were transfected with a replication‐incompetent second‐generation lentivirus expressing the GFP protein, then transfected with two packaging plasmids, one containing gag and pol sequences and regulatory genomic elements (tat and rev), and the second containing vesicular stomatitis virus G (VSV‐G) glycoprotein to give the combined virus particle stability and broader tissue tropism than the human immunodeficiency virus (HIV) membrane that the lentiviral vector was derived from. After transfection, the muscle cell line was expanded and passaged twice to remove any cells critically affected by the random insertion of the viral genome.

### 4.8 Muscle cell line alignment assay

Fibroblasts were plated on 48‐well Nunclon plates at 25,000 cells per well, 6 wells per cell line and incubated in FGF for 1 week followed by fibroblast differentiation media for 2 weeks. Culture plates were decellularised by following a previously described protocol (Franco‐Barraza et al., 2017) and were then seeded with GFP‐transfected HMC25 human muscle cells at 25,000 cells per well, 6 wells per line. Muscle cells were grown to confluence in human muscle growth medium (PromoCell), supplemented with 20% FBS (Gibco, Thermo Fisher) and 50 μg/mL Gentamicin (Gibco, Thermo Fisher) for 6 days. To encourage differentiation into mature myofibres, cells were ‘serum‐starved’ by culturing in human muscle differentiation medium (PromoCell) supplemented with 50 μg/mL gentamicin (Gibco, Thermo Fisher) for 8 days. Wells were then rinsed with DPBS and fixed with 4% PFA for 30 min. Wells were stained and imaged using the previously described immunohistochemistry protocol above for myosin heavy chain (Table 1).

Alignment of the muscle fibres was analysed following the previously described protocol above using a MATLAB script (Cetera et al., 2014).

### OPEN RESEARCH BADGES

This article has earned Open Data and Open Materials badges. Data and materials are available at https://kclpure.kcl.ac.uk/portal/en/persons/malcolm.logan.

## Data Availability

All data will be made available upon reasonable request.
